# Carbohydrate antigen-125 (CA125): a marker of right ventricular dysfunction and poor prognosis in heart failure with preserved ejection fraction

**DOI:** 10.1016/j.ijcha.2025.101775

**Published:** 2025-08-21

**Authors:** Sher May Ng, Geert H.D. Voordes, Michelle Lobeek, Michiel Rienstra, Adriaan A. Voors, Elke S. Hoendermis, Dirk J. van Veldhuisen, Thomas M. Gorter

**Affiliations:** a University of Groningen, University Medical Centre Groningen, Department of Cardiology, Hanzeplein 1, PO Box 30.001, 9700 RB Groningen, the Netherlands; b Oxford Centre for Clinical Magnetic Resonance Research (OCMR), Division of Cardiovascular Medicine, Radcliffe Department of Medicine, University of Oxford, Level 0, John Radcliffe Hospital, Oxford OX3 9DU, United Kingdom

**Keywords:** CA125, Right ventricular dysfunction, HFpEF

## Abstract

**Background:**

Right ventricular (RV) dysfunction (RVD) in heart failure (HF) with preserved ejection fraction (HFpEF) is recognised late and associated with poor outcomes. We aimed to identify biomarkers associated with RV dysfunction in HFpEF and evaluate their prognostic significance.

**Methods:**

77 patients with HFpEF were enrolled from a prospective, multicentre study. At baseline, patients underwent echocardiography, cardiac magnetic resonance (CMR) imaging and laboratory testing. They were followed up for the composite outcome parameter of all-cause mortality and HF hospitalisation. RVD was defined as RV ejection fraction (RVEF) < 45 % on CMR. Proteomics analysis was performed using Olink proteomics multiplex panels (CVDII, CVDIII, Inflammatory and Immuno-oncology) with further verification on immunoassay analysis.

**Results:**

19 patients with HFpEF (25 %) had RVD. The Olink proteomic analysis identified carbohydrate antigen 125 (CA125) as the most differentially abundant in plasma of patients with HFpEF and RVD as compared to those without RVD, which corroborated with further immunoassay analysis − median CA125 in patients with RVD was 23 kU/L [21–47] vs. 16 [[12], [13], [14], [15], [16], [17], [18], [19], [20]] in patients without RVD (p < 0.001). Log-normalised CA125 (LnCA125) was associated with worse RVEF (r = −0.29, p = 0.03) and predicted worse clinical outcomes [HR 2.28 (1.28–4.07) for the composite outcome of all-cause mortality and HF hospitalisation] adjusted for age, gender, body mass index, LVEF, RVD, atrial fibrillation, renal function and NTproBNP.

**Conclusion:**

Targeted proteomic analysis reveals CA125 as a biomarker for RVD in a HFpEF population. Higher serum CA125 concentration, but not NTproBNP, was associated with an increased risk of all-cause mortality and HF hospitalisation.

Word Count: 249.

## 1 Introduction

Right ventricular dysfunction (RVD) in heart failure (HF) with preserved ejection fraction (HFpEF) is prevalent and associated with worse prognosis [1]. Whilst the pathophysiology and chronology of RVD in HFpEF are debated, RV function declines more rapidly than left ventricular (LV) function in HFpEF, with the development of incident RVD associated with a two-fold increased risk of death. [2].

The change in RV morphology and systolic function presents a unique opportunity to monitor disease progression, especially in HFpEF where changes in LV function may be more subtle. Acknowledging this, the Heart Failure Association of the European Society of Cardiology proposed a staging system for RVD encouraging earlier detection, although prospective validation of its clinical significance remains lacking [3]. Additionally, comprehensive assessment of RV structure and function remains challenging with echocardiography. Lack of consensus on prognostically-important parameters and their respective cut-off thresholds for diagnosing RVD further impedes clinical adoption of detailed RV characterisation in the assessment of HFpEF [4,5].

Beyond imaging parameters, there are also no reliable serum biomarkers specifically reflecting RVD in HFpEF. Whilst serum N-terminal pro B-type natriuretic peptide (NTproBNP) is widely used, abnormalities are not specific to RV pathology and values further confounded by additional cardiac and extracardiac comorbidities [6]. This partly reflects the scarcity of clinical trials utilising other biomarkers that may better depict underlying pathophysiology of cardiac remodelling. Diseases with significant heterogeneity such as HFpEF stand to gain from −omics approaches where unknown or unpredicted biomarkers representing disease mechanism may be identified [7]. Bench-to-bedside efforts proposing novel biomarkers specific to RVD in patients with HFrEF [8] and pulmonary hypertension [[9], [10], [11]] with exciting clinical translational potential have been made. However, the complex pathogenesis of RVD in HFpEF may not be adequately captured by these prior studies.

In this study, we performed a comprehensive, multiplex proteomics analysis using panels of selected proteins on a heterogeneous population with HFpEF to identify circulating biomarkers associated with RVD. We then reviewed the association of identified biomarker(s) with common imaging parameters in HFpEF to assess its specificity to RVD. Lastly, the prognostic significance of identified biomarker(s) was assessed by exploring its association with all-cause mortality and HF hospitalisation.

## 2 Methods

### 2.1 Study procedure

Patients included in this study were part of an investigator-initiated, prospective, multicentre study conducted between January 2015 and December 2019 [Ventricular Tachyarrhythmia Detection by Implantable Loop Recording in Patients with Heart Failure and Preserved Ejection Fraction (VIP-HF)]. The inclusion criteria and key exclusion criteria for the VIP-HF study were previously discussed [12]. For this study, only a subgroup of patients with an LVEF ≥50 % and available CMR were included for further analysis. (Supplementary Fig. 1) Echocardiographic evidence of functional or structural alterations [septal/posterior wall thickness ≥11 mm, LV diastolic dysfunction (mean septal/lateral e’ <9 cm/s or E/e’≥13), or left atrial dilatation (left atrial volume index ≥34 ml/m^2^)], or a combination of these parameters were required.

All patients underwent a standard assessment for HF including a comprehensive medical history, physical examination, laboratory testing, echocardiogram and cardiac magnetic resonance imaging (CMR). RVD was defined as a CMR RV ejection fraction (RVEF) < 45 % based on its prognostic significance from a prior study [13].

CMR was performed on a 1.5 Tesla scanner (Philips, Amsterdam, Netherlands and Siemens, Erlangen, Germany) using a standard protocol for the acquisition of cardiac volumes, function and mass. The CMR protocol including typical sequence parameters have been described in detail previously [14]. CMR images were analysed offline by experienced observers using a dedicated software (QMass 7.6 and 8.1, QStrain 2.0, Medis, Leiden, The Netherlands). Longitudinal LV and RV strain measurements were performed on long-axis cine images using the feature/tissue-tracking analysis tool. Atrial volumes and function were estimated using the area-length method on long-axis images. LA and RA reservoir, passive (conduit) and active (booster) strains were assessed using four- and two-chamber long-axis cine images. Right ventricular-pulmonary artery (RV-PA) coupling was assessed by the ratio of RV stroke volume to end-systolic volume on CMR (RV SV/ESV).

The composite outcome of all-cause mortality and first HF hospitalisation was assessed as part of the VIP-HF study. HF hospitalisation was defined as a hospital admission with at least one overnight stay requiring intravenous diuretics or an increased dose of diuretics. Follow-up time was defined as the time from the start of the study to the occurrence of either of the composite endpoints, or the end-of-study visit within 2-years follow-up, whichever occurred first. This study complies with the Declaration of Helsinki. All patients provided written informed consent, and the study was approved by the local medical ethics committee.

### 2.2 Biomarker discovery (proteomics analysis) and validation

77 patients with confirmed LVEF ≥50 % were divided into two groups according to the absence or presence of RVD. Blood specimens were collected, centrifuged and plasma stored at −80 °C. Plasma samples were tested by proximity extension assay (PEA) provided by the Olink Target 96 Cardiovascular disease II, Cardiovascular disease III, Inflammation and Immuno-Oncology panels (Olink Proteomics, Uppsala, Sweden, https://www.olink.com). In the PEA method, pairs of oligonucleotide-labeled antibodies bind to their specific protein targets. Upon binding, the oligonucleotides are brought into proximity, enabling their extension and subsequent quantification via real-time PCR on the Fluidigm BioMark system. The platform provides normalised protein expression rather than absolute quantification [15]. A chemiluminescent microparticle immunoassay (CLIA)-based analysis (Abbott Alinity) on plasma samples was performed to verify findings from the proteomic analysis

### 2.3 Statistical analysis

For the biomarker pathway analysis, proteins were normalised using Olink’s internal reference samples to derive a relative quantification unit, normalised protein expression (NPX). Differential expression analysis using limma (R/Bioconductor software package, version 3.60.3) was performed [16]. Both unadjusted and adjusted (age and gender) analyses were performed. We adjusted for false discovery rate (FDR) using the Benjamini-Hochberg procedure. Proteins with an FDR-adjusted p-value <0.05 were deemed statistically significant. Statistically significant proteins with a log fold-change > 0 were deemed upregulated and proteins with a log fold-change <0 were deemed downregulated.

Continuous data are presented as means ± standard deviation (SD) or medians (interquartile range, IQR). Categorical data are presented as numbers (percentages). Baseline characteristics, imaging parameters and laboratory results were compared using independent *T*-test, Mann-Whitney *U* test, χ^2^ test or Fisher’s exact test according to type and distribution of data. Log-normal transformation was performed for variables when a skewed distribution of residuals was present. Multiple linear regression analysis was used to assess variables associated with biomarker levels and RVEF. Univariable and multivariable Cox proportional hazard regression analyses were performed to assess the associations with clinical outcomes. Covariates included in the multivariable Cox proportional hazard regression analysis were determined by univariable analysis and prior knowledge. The importance of relevant predictor variables was compared by assessing its influence on model fit using the likelihood ratio test. Kaplan-Meier curves were plotted to illustrate the event-free survival of HFpEF patients. An inverse probability of treatment weighting (IPTW) analysis using propensity score matching was also performed to further account for potential confounders. Covariate balance between groups was assessed using standardised mean differences (SMD) with an SMD < 0.1 considered acceptable balance. Statistical analyses were performed using SPSS (version 29, IBM, Armonk, NY) and R (version 4.3.1, Vienna, Austria, https://www.R-project.org).

## 3 Results

### 3.1 Baseline demographics

77 patients had an LVEF ≥ 50 % on echocardiography and were included in this present study. 19 (25 %) patients had evidence of RVD on CMR. Patients with RVD were significantly older but had similar body mass index (BMI), prevalence of atrial fibrillation (AF) and obstructive airway disease to patients without evidence of RVD (no-RVD). A greater proportion of patients in the RVD group had prevalent permanent AF (58 % vs. 21 %, p = 0.023). There was no difference in baseline diuretic therapy, functional status and previous HF hospitalisations between groups. (Table 1)

**Table 1:** Baseline characteristics and CMR parameters.

	**No RVD** **(n = 58)**	**RVD** **(n = 19)**	**p-value**
Age, years (SD)	72 (8)	78 (7)	**0.003**
Female (%)	36 (62)	8 (42)	0.1
BMI, kg/m^2^ (SD)	31.0 (6.1)	29.3 (4.8)	0.3
Systolic BP, mmHg (SD)	145 (21)	135 (23)	0.1
Diastolic BP, mmHg (SD)	73 (14)	72 (16)	0.6

*Comorbidities*			
Hypertension (%)	50 (86)	15 (79)	0.4
Coronary artery disease (%)	21 (36)	5 (26)	0.4
Atrial fibrillation (%)	36 (62)	15 (79)	0.2
Diabetes mellitus (%)	27 (47)	7 (37)	0.5
COPD (%)	9 (16)	4 (21)	0.6

*HF characteristics*			
NYHA functional class			0.3
Class II (%)	32 (55)	8 (42)	
Class III (%)	26 (45)	11 (58)	
Previous HF hospitalization (%)	24 (41)	9 (49)	0.6

*CMR*			
*Left Ventricle*			
EDVi, ml/m^2^ (SD)	86 (23)	78 (21)	0.23
ESVi, ml/m^2^ (SD)	39 (15)	40 (13)	0.84
Ejection fraction, % (SD)	56 (8)	50 (6)	**0.007**
GLS, % (SD)	17 (5)	17 (4)	0.8
Cardiac index, ml/min/m^2^ (SD)	3.2 (0.8)	2.9 (0.5)	**0.04**
*Right Ventricle*			
EDVi, ml/m^2^ (SD)	75 (15)	91 (24)	**0.014**
ESVi, ml/m^2^ (SD)	33 (9)	56 (16)	**<0.001**
Ejection fraction, % (SD)	57 (8)	39 (4)	**<0.001**
GLS, % (SD)	21 (6)	16 (5)	**0.002**
RV SV/ESV (SD)	1.4 (0.6)	0.7 (0.1)	**<0.001**
*Left Atrium*			
LAESVi, ml/m^2^ (SD)	59 (19)	70 (23)	**0.03**
Emptying fraction, % (SD)	31 (16)	16 (9)	**<0.001**
Reservoir strain, % (SD)	15 (9)	8 (4)	**<0.001**
*Right Atrium*			
RAESVi, ml/m^2^ (SD)	39 (17)	60 (24)	**<0.001**
Emptying fraction, % (SD)	32 (16)	17 (11)	**<0.001**
Reservoir strain, % (SD)	23 (15)	9 (6)	**<0.001**

Medication use			
Beta blockers (%)	51 (88)	18 (95)	0.4
ACEi/ARB (%)	36 (62)	13 (68)	0.7
MRA (%)	26 (45)	4 (21)	0.07
Diuretics (%)	51 (88)	19 (100)	0.1

*Laboratory Tests*			
Haemoglobin, mmol/L [IQR]	8.1 [7.2, 8.7]	8.3 [7.4, 9.3]	0.5
Haematocrit, % [IQR]	40 [36, 43]	41 [38, 45]	0.5
C-reactive protein, mg/L [IQR]	3.8 [2.0, 8.4]	4.2 [2.1, 10.0]	0.6
Creatinine, μmol/L [IQR]	100 [83, 131]	127 [106, 154]	**0.02**
eGFR, ml/min/1.73 m^2^ [IQR]	53 [37, 74]	39 [27, 43]	**0.008**
NT-proBNP, ng/L [IQR]	1255 [648, 2234]	2009 [1260, 4595]	**0.01**
AST/ALT [IQR]	1.2 [1.0, 1.4]	1.1 [1.0, 1.4]	0.7

BMI: body mass index; BP: blood pressure; COPD: chronic obstructive pulmonary disease; NYHA: New York Heart Association; LV: left ventricle; RV: right ventricle; EDVi: end-diastolic volume index; ESVi: end-systolic volume index; GLS: global longitudinal strain; RV SV/ESV: right ventricular stroke volume/end-systolic volume ratio; LAESVi: left atrial end-systolic volume index; RAESVi: right atrial end-systolic volume index; ACEi: angiotensin converting enzyme inhibitor; ARB: angiotensin receptor blocker; MRA: mineralocorticoid receptor antagonist; eGFR: estimated glomerular filtration rate; NT-proBNP: N-terminal probrain natriuretic peptide; AST/ALT: aspartate aminotransferase/alanine aminotransferase ratio. Values presented as mean (standard deviation) or median [Q1, Q3].

### 3.2 Cardiac morphology and function

As expected, the RVD group had higher RV volumes with worse longitudinal strain and RV-PA coupling (RV SV/ESV 0.66 ± 0.11 vs. 1.42 ± 0.57, p < 0.001) on CMR. Lower LVEF (RVD 50 ± 6 vs. no-RVD 56 ± 8 %, p = 0.007) and cardiac index (2.9 ± 0.5 vs. 3.2 ± 0.8 L/min/m^2^, p = 0.04) on CMR was also observed as compared to the no-RVD group. The RVD group had worse biatrial function as measured by atrial emptying fraction and reservoir strain. There was no difference in estimated pulmonary capillary wedge pressure (PCWP) by E/e’ on echocardiography between groups (Supplementary Table 1).

### 3.3 Circulating biomarkers associated with RV dysfunction in HFpEF

**Fig. 1:**
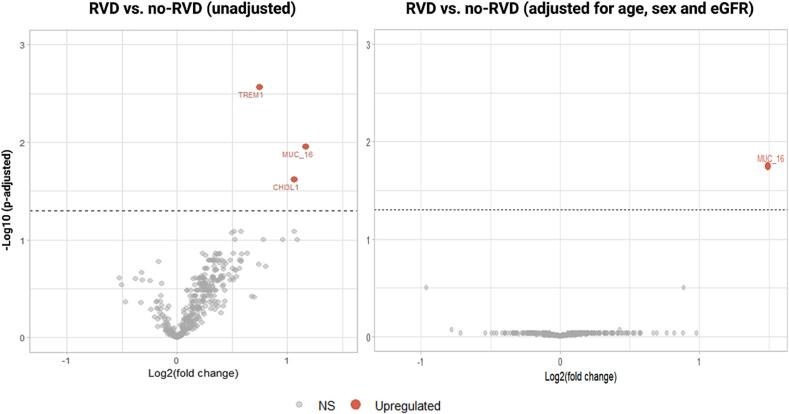
Olink proteomic analysis. Volcano plots depicting relative abundances of plasma proteins in HFpEF patients with RVD vs. no RVD (A) unadjusted (B) adjusted for age, sex and estimated glomerular filtration rate (eGFR). MUC_16 (CA125) is significantly upregulated with a log2 fold change of 1.5 (p = 0.003).

The relative abundances of 367 proteins in the plasma of HFpEF patients with and without RVD were determined using Olink Target 96 proteomic analyses. In an unadjusted analysis, carbohydrate antigen-125 [CA125 (MUC_16)], triggering receptor expressed on myeloid cells-1 (TREM-1) and chitinase-3 like protein 1 (CHI3L) were upregulated in the RVD group as compared to the no-RVD group. However, only CA125 was differentially increased in the RVD-HFpEF cohort (∼2.8-fold, p = 0.003) when adjusted for age, sex and renal function. (Fig. 1) NTproBNP was not differentially expressed in either unadjusted or adjusted analyses. The complete list of analysed proteins is available in Supplementary Table 2.

In the same cohort, plasma concentration of CA125 measured by CLIA was significantly higher in the RVD-HFpEF group [median 23 kU/L [21, 47] vs. 16 (12, 20), p < 0.001; Supplementary Fig. 2A]. Both groups had elevated plasma NTproBNP levels, with significantly higher values in the group with RVD (Table 1).

### 3.4 Relationship between CA125 and cardiac function in HFpEF

Log-normalised CA125 levels (lnCA125) correlated with RVEF on CMR, adjusting for age, BMI, underlying AF diagnosis, renal function, LVEF and diuretic use (r = −0.29, p = 0.03; Supplementary Fig. 2B). Higher CA125 levels negatively correlated with RV-PA coupling, irrespective of underlying AF [r = −0.25, p = 0.04], although this was no longer significant when accounting for RVEF.

No association was observed between lnCA125 and LVEF or renal function (as measured by estimated glomerular filtration rate, eGFR). (Supplementary Table 3) Whilst there was moderate correlation between lnNTproBNP and lnCA125 (r = 0.42, p < 0.001), no association was observed between lnNTproBNP and RVEF on multivariable linear regression analysis (Supplementary Table 4). LA emptying fraction (r = −0.31, p = 0.01) and reservoir function (r = −0.28, p = 0.02), but not maximal LA volume (r = 0.19, p = 0.12) were negatively correlated with lnCA125, even when accounting for age, gender, BMI and underlying AF.

Receiver operator curve (ROC) analysis was performed to compare the predictive capacity of CA125 and NTproBNP for RVD in HFpEF. The area under the curve (AUC) for CA125 was 0.82 (95 % CI 0.71 – 0.93), numerically higher than NTproBNP – 0.70 (95 % CI 0.55 – 0.85). Using Youden’s index on ROC analysis, a plasma CA125 level of 19.5 kU/L had a sensitivity of 87 % and specificity of 71 % for RVD in HFpEF (Supplementary Fig. 3).

### 3.5 CA125 and clinical outcomes in HFpEF

The combined outcome of all-cause death and HF hospitalisation occurred in 25 patients (32 %) over a median follow-up of 24 months (17–26). 42 % of patients with RVD (8/19) as compared to 29 % of patients without RVD (17/58) reached the combined outcome (p = 0.30). All-cause death occurred in 15 [6 RVD (32 %), 9 no-RVD (16 %); p = 0.13) and HF hospitalisation in 19 (5 RVD (26 %), 14 no-RVD (24 %); p = 0.85) patients, respectively. 9 patients (12 % of overall cohort) experienced both events.

On Cox’s proportional hazards regression model analysis including age, gender, BMI, LVEF, RVD, AF, LAEF, lnCA125, lnNTproBNP and renal function (log-normalised eGFR, lneGFR), only lnCA125 levels, BMI and renal function were associated with the combined outcome of all-cause death and hospitalisation [lnCA125 HR 2.28 (1.28–4.07), p = 0.005; BMI HR 1.14 (1.03–1.27), p = 0.01; lneGFR HR 0.23 (0.07–0.71), p = 0.01]. (Table 2) LnCA125 was also independently associated with all-cause mortality [HR 2.29 (1.40–3.76), p < 0.001]. The removal of lnCA125 from the multivariable Cox regression model resulted in the greatest change in Akaike information criterion (AIC) and χ^2^ value on likelihood ratio test (p = 0.002, Supplementary Table 5). Whilst some studies suggest higher CA125 concentrations predict decline in renal function [17], there was no significant correlation between lnCA125 and baseline eGFR in our cohort when adjusted for age, gender and BMI (r = −0.23, p = 0.07).

**Table 2:** Cox’s proportional hazards regression model for combined endpoint of all-cause death and HF hospitalisation.

	**Univariable**		**Multivariable**	
Age	1.02 [0.97–1.07]	p = 0.38		
Sex	1.21 [0.55–2.65]	p = 0.64		
BMI	1.08 [1.00–1.15]	**p = 0.04**	1.14 [1.03–1.27]	**p = 0.01**
AF	0.77 [0.34 – 1.71]	p = 0.51		
RV dysfunction (RVEF < 45 %)	1.46 [0.63–3.39]	p = 0.38		
LVEF	1.01 [0.95–1.06]	p = 0.86		
LneGFR	0.10 [0.04–0.28]	**p < 0.001**	0.23 [0.07–0.71]	**p = 0.01**
LnNTproBNP	1.72 [1.05–2.81]	**p = 0.03**	NS	
LnCA125	2.03 [1.33–3.10]	**p = 0.001**	2.28 [1.28–4.07]	**p = 0.005**
LAEF	0.98 [0.96–1.01]	P = 0.16		

BMI: body mass index (continuous); RVD: right ventricular dysfunction (RVEF < 45 % on CMR); LVEF: left ventricular ejection fraction (continuous); LneGFR: log-normalised estimated glomerular filtration rate; LnNTproBNP: log-normalised N-terminal probrain natriuretic peptide; LnCA125: log-normalised carbohydrate antigen 125. LAEF: left atrial emptying fraction.

An inverse probability of treatment weighting (IPTW) analysis using propensity score matching was also performed as an alternative method to adjust for potential confounders. Propensity scores were estimated using logistic regression including age, sex, BMI, AF, history of coronary artery disease, diabetes mellitus, previous heart failure hospitalisation, diuretic use, renal function, RV dysfunction, LVEF and lnNTproBNP as covariates. Stabilised weights were used to reduce variance. The model ultimately included 66 patients with 22 events. In the weighted Cox’s proportional hazards regression model, an elevated serum CA125 (above median) was significantly associated with an increased risk of the combined endpoint of all-cause mortality and HF hospitalisation [weighted HR 3.54 (1.38–9.07), p = 0.008].

A few covariates (RV dysfunction, LVEF, lnNTproBNP, previous hospitalisation and diuretic use) remained imbalanced (SMD > 0.10) suggesting unadjusted confounders. To further minimise model misspecification, a doubly robust estimation combining the IPTW model and imbalanced covariates was used. In this model, diuretic use was excluded due to severe separation or near-perfect prediction, indicating the expected association between congestion and impending HF hospitalisation. A trend towards higher risk of all-cause mortality or HF hospitalisation was observed with higher CA125 levels [HR 2.7 (0.91–8.0), p = 0.07], although this did not meet statistical significance.

**Fig. 2:**
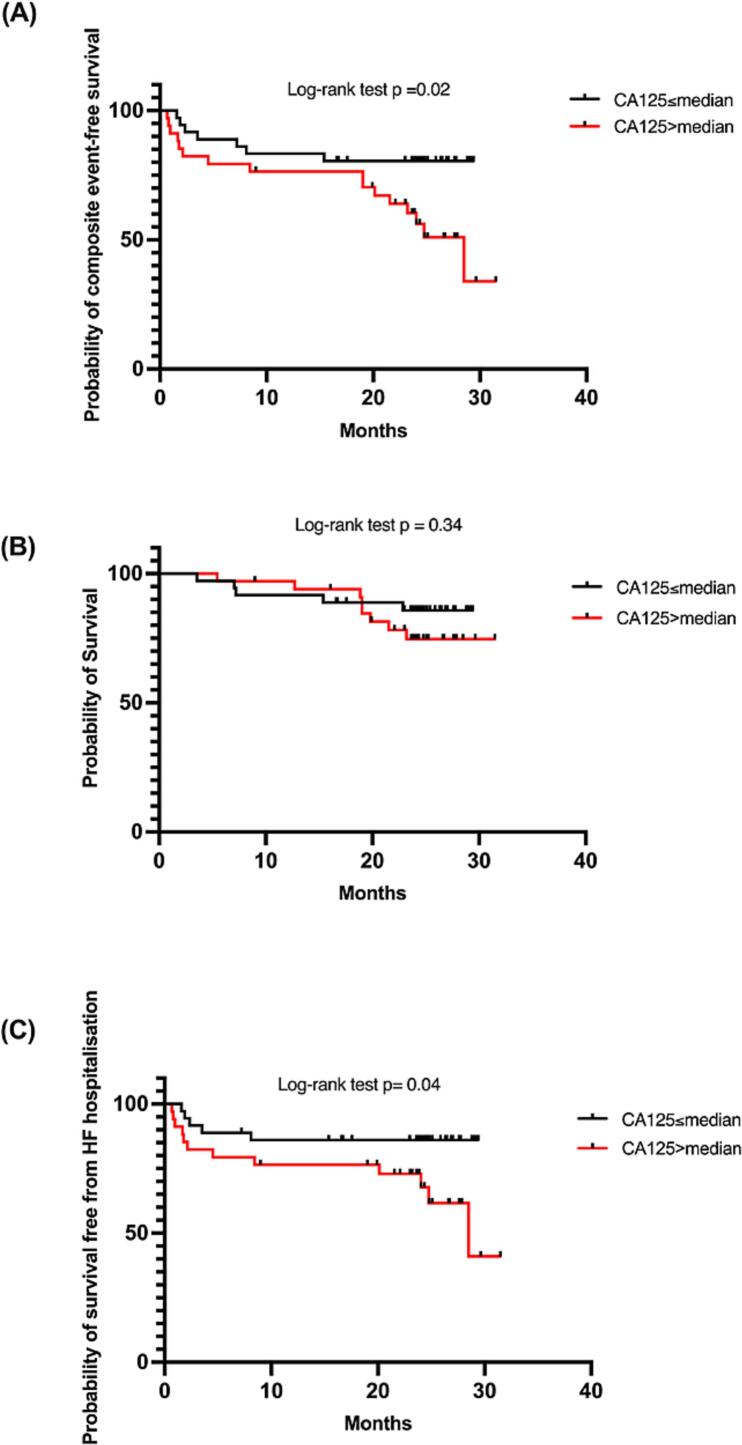
(A) Probability of combined event-free survival (B) all-cause mortality and (C) heart failure hospitalisation by cohort median CA125 (17kU/L).

Consistent with this, patients with CA125 levels above the cohort median (17kU/L) had a significantly lower probability of survival free from the combined endpoint of all-cause mortality or HF hospitalization, but not from all-cause mortality alone. (Fig. 2) The group with higher CA125 levels also had higher C-reactive protein and NTproBNP levels, with a greater proportion reporting NYHA class III symptoms (Supplementary Table 6). A ROC analysis of plasma CA125 levels for the combined endpoint of all-cause death and HF hospitalisation was of modest quality (AUC 0.70, p = 0.003), demonstrating a value of 17.5kU/L to be the optimal cut-off value for worse clinical outcomes.

## 4 Discussion

Our study highlights several important points in a prospectively recruited HFpEF population: (1) RVD as assessed by gold-standard CMR quantification is common in HFpEF, affecting approximately 25 % of our study cohort. (2) Through comprehensive proteomic analysis in a HFpEF population with elevated NTproBNP, CA125 is differentially upregulated in patients with evidence of RVD. (3) CA125 is independently associated with RVD and adverse clinical outcomes in HFpEF, despite adjusting for age, biventricular function, AF diagnosis and LA function, renal function as well as NTproBNP.

CA125 is recognised as a marker of congestion and an independent predictor of outcomes in certain HF subpopulations. A large glycoprotein synthesised by mesothelial cells on serosal surfaces such as the pericardium, pleura and peritoneum, its upregulation in context of decompensated HF is thought to be in response to increased hydrostatic pressure, mechanical stress and cytokine activation [18]. However, these remain speculative, and the underlying mechanism of increased CA125 expression i.e. whether the upregulation simply represents a consequence of congestion or a trigger for decompensation, remains debated [19].

Prior studies in patients with HF with reduced ejection fraction (HFrEF) suggest CA125 is more closely associated with features of congestion (e.g. severity of pleural effusion and tricuspid regurgitation) [20]. Few have demonstrated the direct correlation between CA125 and RV function assessed on CMR, although elevated CA125 levels have been described in case reports of isolated RV failure due to an atrial septal defect [21] and chronic obstructive pulmonary disease [22]. Our observation that CA125 but not NTproBNP was differentially upregulated in patients with HFpEF and RV dysfunction raises the possibility CA125 more closely reflects RV impairment beyond that of NTproBNP, where increased expression particularly in circumstances of fluid overload, may be expected in HFpEF. The persistence of an association between CA125 and RVEF after accounting for diuretic use in our cohort may suggest that this relationship extends beyond the influence of congestion. The absence of association between LV systolic (LVEF) and diastolic (E/e’) function with CA125 levels in our HFpEF cohort may further support the specificity of CA125 as a marker of RVD. Nevertheless, cautious interpretation of serum CA125 concentration is required due to its lack of organ or disease specificity.

Concurrent AF with HFpEF has also been independently associated with worse RV function.(2) Whilst there was greater prevalence of permanent AF in HFpEF patients with RVD in our cohort, the association between higher CA125 levels and worse RV function as well as RV-PA coupling was independent of underlying AF. This supports the potential role for CA125 as a marker of RV compromise in HFpEF disease progression, regardless of underlying cardiac rhythm [23].

Whether AF affects serum CA125 concentrations remains unclear; whilst we observed no association between underlying AF diagnosis with CA125 levels in our small cohort, studies in patients with idiopathic AF and HFrEF suggest higher CA125 levels in patients with paroxysms of AF as compared to sinus rhythm [24,25]. Conversely, others propose higher baseline CA125 levels in sinus rhythm is associated with an increased risk of new-onset AF, suggesting it could reflect underlying pathophysiological processes driving atrial myopathy [26,27]. Indeed, the latter may be in keeping with our observation of worse LA function with higher CA125 levels, independent of the presence or absence of AF. It is therefore plausible that elevated CA125 levels represent impending left atrial dysfunction, which subsequently mediates the development of RV dysfunction through alterations in pulmonary vascular pressure.

Studies proposing the use of CA125 to identify RVD in HFpEF are scarce. Here, we demonstrate plasma CA125 levels had superior predictive capacity of worse RV function in HFpEF as compared to NTproBNP. Our findings are in line with a recent prospective study demonstrating a higher proportion of HFpEF patients with RVD in a group with CA125 levels above 35kU/L [28]. Our data also supports the growing body of evidence demonstrating the added value of CA125, on top of NTproBNP at predicting the composite endpoint of all-cause mortality and HF hospitalisation in HFpEF. Two potential explanations include: [1] CA125 closely reflects congestion status and higher levels may portend imminent decompensation and therefore, HF hospitalisation. The association between worse renal function and adverse clinical outcomes further supports this notion (2) Beyond RV dysfunction, higher CA125 levels may suggest more advanced HFpEF and thus worse prognosis. This would be in keeping with observations of worse LA function and RV-PA coupling, regardless of the underlying cardiac rhythm.

Additionally, we demonstrate a lower CA125 cut-off value of 17kU/L (cohort median) instead of the conventional cut-off value of 35kU/L for suspected ovarian malignancy predicted worse clinical outcomes. A large prospective study of recently hospitalised patients with HFpEF similarly demonstrated patients with a median CA125 level of 26.8 [22.5–32] were already at an increased risk of all-cause mortality and recurrent HF admissions, prompting the need for a different CA125 cut-off threshold in the HF realm [29]. Similar to Menghoum and Miñana et al., we did not observe an additive value of NTproBNP at predicting composite endpoints in our HFpEF population.

## 5 Limitations

A limitation of our study is the lack of objective assessment of congestion status. This makes it challenging to distinguish whether the prognostic value of CA125 observed is simply a consequence of volume status. CA125 but not NTproBNP remained significantly associated with adverse outcomes despite the correlation between CA125 and NTproBNP, also a marker of congestion. This suggests the prognostic significance of CA125 may extend beyond its reflection of fluid status. Using the AST/ALT ratio and haematocrit levels as surrogates of congestion, we observed no difference between groups according to RV function or median CA125 levels. Nevertheless, there was a numerically higher percentage of patients with CA125 levels above the cohort median on diuretic therapy, although this was not statistically significant.

This was a small study with only 32 % of the cohort reaching the composite primary endpoint of all-cause death or HF hospitalisation. These findings are therefore hypothesis-generating and require further validation with larger studies. The cross-sectional nature of our study further hampers our ability to delineate the longitudinal relationship between CA125 and RV function in HFpEF. Patients were recruited over a study period of 2015 to 2019 where understanding of the diagnosis and management of HFpEF remained at its infancy. In particular, this study occurred before the introduction of SGLT2 inhibitors as part of HFpEF therapy and is therefore unable to comment on the influence of SGLT2 inhibitors on CA125 levels and RV function.

We acknowledge the limited availability of cardiac MRI globally for accurate RV assessment. As our study is limited by a small sample size and a significant proportion of missing echocardiographic measurements relevant to RV function (23–38 %), larger studies identifying prognostically-relevant thresholds for echocardiographic parameters and their association with CA125 should be considered. Dedicated HFpEF studies assessing serial CA125 levels in conjunction with RV imaging parameters and clinical outcomes will allow better understanding of the reliability of CA125 as a biomarker for RV function in HFpEF.

## 6 Conclusion

CA125 is differentially expressed in HFpEF individuals with concurrent RV dysfunction as compared to HFpEF individuals with preserved RV function on a comprehensive proteomics approach. We demonstrate that CA125 is superior to NTproBNP and predicts RV dysfunction in a multimorbid HFpEF cohort. Importantly, our findings support increasing evidence that CA125 is independently associated with adverse clinical outcomes in a HFpEF population. Future studies confirming the role of CA125 as a biomarker of RV deterioration in HFpEF and its complementary role in HFpEF phenotyping and prognosis will be of great interest.

## Authorship statement

The authors take responsibility for all aspects of the reliability and freedom from bias of the data presented and their discussed interpretation

## Funding

The VIP-HF study was supported by an unrestricted grant from Abbott-Netherlands to the University Medical Centre Groningen. Abbott-Netherlands was neither involved in the conduction of the study, nor in the writing of this article.

## CRediT authorship contribution statement

**Sher May Ng:** Writing – original draft, Visualization, Investigation, Formal analysis, Data curation, Conceptualization. **Geert H.D. Voordes:** Writing – review & editing, Visualization, Software, Methodology, Investigation, Formal analysis, Data curation. **Michelle Lobeek:** Writing – review & editing, Project administration, Methodology, Investigation, Data curation. **Michiel Rienstra:** Writing – review & editing, Validation, Supervision, Resources, Project administration, Funding acquisition. **Adriaan A. Voors:** Writing – review & editing, Validation, Supervision, Resources. **Elke S. Hoendermis:** Writing – review & editing, Supervision, Resources. **Dirk J. van Veldhuisen:** Writing – review & editing, Supervision, Resources, Project administration, Conceptualization. **Thomas M. Gorter:** Writing – original draft, Supervision, Project administration, Methodology, Investigation, Funding acquisition, Formal analysis, Data curation, Conceptualization.

## Declaration of competing interest

The authors declare the following financial interests/personal relationships which may be considered as potential competing interests: Michiel Rienstra reports financial support was provided by Abbott-Netherlands. Michiel Rienstra reports a relationship with Netherlands Organisation for Health Research and Development that includes: funding grants. Michiel Rienstra reports a relationship with Dutch Heart Foundation that includes: funding grants. Michiel Rienstra reports a relationship with Netherlands Cardiovascular Research Initiative that includes: funding grants. Michiel Rienstra reports a relationship with Top Sector Life Sciences & Health to the Dutch Heart Foundation that includes: funding grants. Thomas Gorter reports a relationship with University Medical Centre Groningen that includes: funding grants. Thomas Gorter reports a relationship with Daiichi Sankyo Inc that includes: speaking and lecture fees. Thomas Gorter reports a relationship with Corvia Medical that includes: consulting or advisory. Sher May Ng reports a relationship with Welcome Trust Clinical Research Training Fellowship that includes: funding grants. Michiel Rienstra reports a relationship with Bayer AG that includes: consulting or advisory. Michiel Rienstra reports a relationship with InCarda Therapeutics, Inc. that includes: consulting or advisory. If there are other authors, they declare that they have no known competing financial interests or personal relationships that could have appeared to influence the work reported in this paper.
