# Supplementary material for: Carbohydrate antigen-125 (CA125): a marker of right ventricular dysfunction and poor prognosis in heart failure with preserved ejection fraction
Source: Int J Cardiol Heart Vasc. 2025 Aug 21;60:101775. doi: 10.1016/j.ijcha.2025.101775 (PMC12490575; doi:10.1016/j.ijcha.2025.101775)
Supplement: Supplementary Data 1 [file mmc1.docx]

CARBOHYDRATE ANTIGEN-125 (CA125): A MARKER OF RIGHT VENTRICULAR DYSFUNCTION AND POOR PROGNOSIS IN HEART FAILURE WITH PRESERVED EJECTION FRACTION

SUPPLEMENTARY MATERIAL

**Supplementary Figure 1:** Flowchart of patient subgroup from Ventricular tachyarrhythmia detection by Implantable loop recording in Patients with heart failure and preserved ejection fraction (VIP-HF) study

Key exclusion criteria:

- ICD or pacemaker
- Indication for ICD therapy
- Life expectancy < 1 year
- MI, PCI or CABG in the last 3 months
- Complex congenital heart disease

**N = 113 recruited into VIP-HF**

*Inclusion criteria:*

*Age > 18 years*

*NYHA II-III*

*Prior hospitalisation, urgent visit or diuretic use for HF in last 12 months*

*NTproBNP>300pg/ml (sinus) or >900 (AF)*

*LVEF>40%*

*Structural/functional alteration on echo:
-septal/posterior wall thickness ≥ 11mm OR*

*-septal/lateral e’ <9cm/s or E/e≥13 OR*

*-LAVI≥34ml/m^2^*

**CMR RVEF < 45%**

No

Yes

**RVD**

**N=19**

**No RVD**

**N=58**

28 LVEF<50% therefore excluded from present study

**N = 77 included in this study**

8 excluded (no cardiac MRI)

**N = 105 underwent cardiac MRI**

**Supplementary table 1:** Baseline echocardiography parameters

|  | **No RVD**  **(n=58)** | **RVD**  **(n=19)** | **p-value** |
| --- | --- | --- | --- |
| LV ejection fraction, % (SD) | 57 (5) | 54 (4) | **0.03** |
| LV mass index, g/m^2^ (SD) | 103 (42) | 105 (28) | 0.8 |
| E/e’ (SD) | 13.8 (5.2) | 11.1 (0.1) | 0.5 |
| Mean e’ sept/lateral wall, cm/s (SD) | 7.5 (2.0) | 8.5 (2.3) | 0.1 |
| LA volume index, ml/m^2^ (SD) | 44 (15) | 52 (10) | **0.04** |
| TAPSE, mm (SD)* | 21 (4) | 18 (4) | 0.05 |
| RV FAC, % (SD)** | 49 (11) | 41 (12) | **0.04** |
| RV S’, cm/s (SD)*** | 11.6 (3.2) | 10.3 (1.7) | 0.06 |
| RV end-diastolic area, cm^2^ (SD) | 14 (5) | 23 (9) | **0.008** |
| SPAP, mmHg (SD) | 41 (16) | 35 (9) | 0.2 |
| TAPSE/SPAP, mm/mmHg (SD) | 0.65 (0.37) | 0.55 (0.20) | 0.4 |

**18 missing values*

***29 missing values*

**** 27 missing values*

*LV: left ventricle; LA: left atrium TAPSE: tricuspid annular plane systolic excursion; RV: right ventricle; FAC: fractional area change; SPAP: pulmonary artery systolic pressure. Mean values and SD presented.*

**Supplementary Table 2:** List of proteins analysed using Olink Target 96 Cardiovascular Disease II, Cardiovascular Disease III, Inflammation and Immuno-Oncology panels

| **Gene** | **Protein name** |
| --- | --- |
| ABL1 | ABL proto-oncogene 1, non-receptor tyrosine kinase |
| ACE2 | Angiotensin-converting enzyme 2 |
| ADAM_TS_15 | ADAM metallopeptidase with thrombospondin type 1 motif 15 |
| ADAM_TS13 | A disintegrin and metalloproteinase with thrombospondin motifs 13 |
| ADAM8 | ADAM metallopeptidase domain 8 |
| ADM | Pro-adrenomedullin |
| AGRP | Agouti-related protein |
| ALCAM | CD166 antigen |
| AMBP | Protein AMBP |
| ANGPT1 | Angiopoietin-1 |
| ANXA1 | Annexin A1 |
| AP_N | Aminopeptidase N |
| AREG_immunepanel | Amphiregulin (immunepanel) |
| AREG_Oncopanel | Amphiregulin (oncopanel) |
| ARNT | Aryl hydrocarbon receptor nuclear translocator |
| AXL | Tyrosine-protein kinase receptor UFO |
| AZU1 | Azurocidin |
| BACH1 | BTB and CNC homology 1, basic leucine zipper transcription factor 1 |
| BIRC2 | Baculoviral IAP repeat containing 2 |
| BLM_hydrolase | Bleomycin hydrolase |
| BMP_6 | Bone morphogenetic protein 6 |
| BNP | B-type natriuretic peptide |
| BOC | Brother of CDO |
| BTN3A2 | Butyrophilin subfamily 3 member A2 |
| CA5A | Carbonic anhydrase 5A, mitochondrial |
| CAIX | Carbonic anhydrase IX |
| CASP_3 | Caspase-3 |
| CCL11 | Eotaxin |
| CCL15 | C-C motif chemokine 15 |
| CCL16 | C-C motif chemokine 16 |
| CCL17 | C-C motif chemokine 17 |
| CCL24 | C-C motif chemokine 24 |
| CCL3 | C-C motif chemokine 3 |
| CD160 | CD160 antigen |
| CD163 | Scavenger receptor cysteine-rich type 1 protein M130 |
| CD207 | Langerin |
| CD27 | CD27 antigen |
| CD28 | T-cell-specific surface glycoprotein CD28 |
| CD4 | T-cell surface glycoprotein CD4 |
| CD40_L | CD40 ligand (CD40L) |
| CD48 | CD48 antigen |
| CD70 | CD70 antigen |
| CD83 | CD83 antigen |
| CD84 | SLAM family member 5 |
| CD93 | Complement component C1q receptor |
| CDH5 | Cadherin-5 |
| CDKN1A | Cyclin-dependent kinase inhibitor 1A (p21, Cip1) |
| CDSN | Corneodesmosin |
| CEACAM1 | Carcinoembryonic antigen-related cell adhesion molecule 1 |
| CEACAM5 | Carcinoembryonic antigen-related cell adhesion molecule 5 |
| CEACAM8 | Carcinoembryonic antigen-related cell adhesion molecule 8 |
| CHI3L1 | Chitinase-3-like protein 1 |
| CHIT1 | Chitotriosidase-1 |
| CKAP4 | Cytoskeleton-associated protein 4 |
| CLEC4A | C-type lectin domain family 4 member A |
| CLEC4C | C-type lectin domain family 4 member C (BDCA-2) |
| CLEC4D | C-type lectin domain family 4 member D |
| CLEC4G | C-type lectin domain family 4 member G |
| CLEC6A | C-type lectin domain family 6 member A |
| CLEC7A | Dectin-1 (C-type lectin domain family 7 member A) |
| CNTN1 | Contactin-1 |
| CNTNAP2 | Contactin-associated protein-like 2 |
| COL1A1 | Collagen alpha-1(I) chain |
| CPA1 | Carboxypeptidase A1 |
| CPB1 | Carboxypeptidase B |
| CPE | Carboxypeptidase E |
| CRNN | Cornulin |
| CSTB | Cystatin-B |
| CTRC | Chymotrypsin-C |
| CTSD | Cathepsin D |
| CTSL1 | Cathepsin L1 |
| CTSV | Cathepsin V |
| CTSZ | Cathepsin Z |
| CXADR | Coxsackievirus and adenovirus receptor |
| CXCL1 | Growth-regulated alpha protein |
| CXCL12 | Stromal cell-derived factor 1 |
| CXCL13 | C-X-C motif chemokine 13 |
| CXCL16 | C-X-C motif chemokine 16 |
| CXL17 | Chemokine-like protein 17 |
| CYR61 | Cysteine-rich angiogenic inducer 61 |
| DAPP1 | Dual adaptor of phosphotyrosine and 3-phosphoinositides |
| DCBLD2 | Discoidin, CUB, and LCCL domain-containing protein 2 |
| DCN | Decorin |
| DCTN1 | Dynactin subunit 1 |
| DDX58 | DEAD-box helicase 58 (RIG-I) |
| DECR1 | 2,4-dienoyl-CoA reductase, mitochondrial |
| DFFA | DNA fragmentation factor subunit alpha |
| DGKZ | Diacylglycerol kinase zeta |
| Dkk_1 | Dickkopf wnt signaling pathway inhibitor 1 |
| DLK_1 | Protein delta homolog 1 |
| DLL1 | Delta-like protein 1 |
| DPP10 | Dipeptidyl peptidase-like protein 10 |
| EDAR | Ectodysplasin-A receptor |
| EGF | Pro-epidermal growth factor |
| EGFR | Epidermal growth factor receptor |
| EGLN1 | Egl-9 family hypoxia-inducible factor 1 |
| EIF4G1 | Eukaryotic translation initiation factor 4 gamma 1 |
| EIF5A | Eukaryotic translation initiation factor 5A |
| Ep_CAM | Epithelial cell adhesion molecule |
| EPHA2 | Ephrin type-A receptor 2 |
| EPHB4 | Ephrin type-B receptor 4 |
| ERBB2 | Receptor tyrosine-protein kinase erbB-2 |
| ERBB3 | Receptor tyrosine-protein kinase erbB-3 |
| ERBB4 | Receptor tyrosine-protein kinase erbB-4 |
| ESM_1 | Endothelial cell-specific molecule 1 |
| FABP2 | Fatty acid-binding protein, intestinal |
| FABP4 | Fatty acid-binding protein, adipocyte |
| FADD | Fas-associated protein with death domain |
| FAM3B | Family with sequence similarity 3 member B (also known as PANDER) |
| FAS | Tumor necrosis factor receptor superfamily member 6 |
| FASLG | Tumor necrosis factor ligand superfamily member 6 |
| FCRL3 | Fc receptor-like protein 3 |
| FCRL6 | Fc receptor-like protein 6 |
| FCRLB | Fc receptor-like B |
| FGF_21 | Fibroblast growth factor 21 |
| FGF_23 | Fibroblast growth factor 23 |
| FGF_BP1 | Fibroblast growth factor-binding protein 1 |
| FGF2 | Fibroblast growth factor 2 |
| FR_alpa | Folate receptor alpha |
| FR_gamma | Folate receptor gamma |
| FS | Follistatin |
| FURIN | Furin, paired basic amino acid cleaving enzyme |
| FXYD5 | FXYD domain-containing ion transport regulator 5 |
| Gal_1 | Galectin-1 |
| Gal_3 | Galectin-3 |
| Gal_4 | Galectin-4 |
| Gal_9 | Galectin-9 |
| GALNT3 | Polypeptide N-acetylgalactosaminyltransferase 3 |
| GDF_15 | Growth/differentiation factor 15 |
| GDF_2 | Growth/differentiation factor 2 |
| GH | Growth hormone |
| GIF | Gastric intrinsic factor |
| GLB1 | Beta-galactosidase |
| GLO1 | Lactoylglutathione lyase |
| GP6 | Platelet glycoprotein VI |
| GPC1 | Glypican-1 |
| GPNMB | Glycoprotein non-metastatic melanoma protein B |
| GRN | Progranulin |
| GZMB | Granzyme B |
| GZMH | Granzyme H |
| HAOX1 | Hydroxyacid oxidase 1 |
| HB_EGF | Heparin-binding EGF-like growth factor |
| HCLS1 | Hematopoietic cell-specific Lyn substrate 1 |
| HEXIM1 | Hexamethylene bisacetamide inducible 1 |
| HGF | Hepatocyte growth factor |
| hK11 | Kallikrein-11 |
| hK14 | Kallikrein-14 |
| hK8 | Kallikrein-8 |
| HNMT | Histamine N-methyltransferase |
| HO_1 | Heme oxygenase 1 |
| hOSCAR | Osteoclast-associated receptor |
| HSD11B1 | Hydroxysteroid 11-beta dehydrogenase 1 |
| HSP_27 | Heat shock protein beta-1 |
| ICA1 | ICA1 antigen |
| ICAM_2 | Intercellular adhesion molecule 2 |
| ICOSLG | ICOS ligand |
| IDUA | Alpha-L-iduronidase |
| IFN_gamma_R1 | Interferon gamma receptor 1 |
| IFNLR1 | Interferon lambda receptor 1 |
| IGF1R | Insulin-like growth factor 1 receptor |
| IGFBP_1 | Insulin-like growth factor binding protein 1 |
| IGFBP_2 | Insulin-like growth factor binding protein 2 |
| IGFBP_7 | Insulin-like growth factor binding protein 7 |
| IgG_Fc_receptor_II_b | IgG Fc receptor IIb |
| IL_17D | Interleukin 17D |
| IL_17RA | Interleukin 17 receptor A |
| IL_18BP | Interleukin 18 binding protein |
| IL_1ra | Interleukin-1 receptor antagonist |
| IL_1RT1 | Interleukin-1 receptor type 1 |
| IL_1RT2 | Interleukin-1 receptor type 2 |
| IL_27 | Interleukin 27 |
| IL_4RA | Interleukin-4 receptor alpha |
| IL_6RA | Interleukin-6 receptor alpha |
| IL10 | Interleukin-10 |
| IL12RB1 | Interleukin-12 receptor subunit beta-1 |
| IL16 | Pro-interleukin-16 |
| IL18 | Interleukin-18 |
| IL1RL2 | Interleukin-1 receptor-like 2 |
| IL2_RA | Interleukin-2 receptor alpha |
| IL5 | Interleukin-5 |
| IL6_CVDII | Interleukin 6 (CVDII) |
| IL6_immunepanel | Interleukin-6 (immunepanel) |
| IL6_oncopanel | Interleukin-6 (Oncopanel) |
| IRAK1 | Interleukin-1 receptor-associated kinase 1 |
| IRAK4 | Interleukin-1 receptor-associated kinase 4 |
| IRF9 | Interferon regulatory factor 9 |
| ITGA11 | Integrin alpha-11 |
| ITGA6 | Integrin alpha-6 |
| ITGAV | Integrin alpha-V |
| ITGB1BP2 | Integrin beta-1-binding protein 2 |
| ITGB2 | Integrin beta-2 |
| ITGB5 | Integrin beta-5 |
| ITGB6 | Integrin beta-6 |
| ITM2A | Integral membrane protein 2A |
| JAM_A | Junctional adhesion molecule A |
| JUN | Jun proto-oncogene |
| KIM1 | Kidney injury molecule-1 |
| KLK13 | Kallikrein-related peptidase 13 |
| KLK6 | Kallikrein-6 |
| KLRD1 | Natural killer cells antigen CD94 |
| KPNA1 | Karyopherin subunit alpha-1 |
| KRT19 | Keratin 19 |
| LAG3 | Lymphocyte activation gene 3 protein |
| LAMP3 | Lysosome-associated membrane glycoprotein 3 |
| LDL_receptor | Low-density lipoprotein receptor |
| LEP | Leptin |
| LILRB4 | Leukocyte immunoglobulin-like receptor B4 |
| LOX_1 | Lectin-like oxidized low-density lipoprotein receptor-1 |
| LPL | Lipoprotein lipase |
| LTBR | Tumor necrosis factor receptor superfamily member 3 |
| LY75 | Lymphocyte antigen 75 |
| LY9 | Lymphocyte antigen 9 |
| LYN | LYN proto-oncogene, Src family tyrosine kinase |
| LYPD3 | Ly6/PLAUR domain-containing protein 3 |
| MAD_homolog_5 | MAD homolog 5 (Mothers against decapentaplegic homolog 5) |
| MARCO | Macrophage receptor MARCO |
| MASP1 | Mannan-binding lectin serine peptidase 1 |
| MB | Myoglobin |
| MCP_1 | Monocyte chemoattractant protein 1 |
| MEPE | Matrix extracellular phosphoglycoprotein |
| MERTK | Tyrosine-protein kinase Mer |
| MetAP_2 | Methionine aminopeptidase 2 |
| MGMT | O-6-methylguanine-DNA methyltransferase |
| MIA | Melanoma inhibitory activity |
| MIC_A_B | MHC class I polypeptide-related sequence A and B |
| MILR1 | Monocyte immunoglobulin-like receptor 1 |
| MK | Midkine |
| MMP_2 | Matrix metallopeptidase 2 (Gelatinase A) |
| MMP_3 | Matrix metallopeptidase 3 (Stromelysin-1) |
| MMP_9 | Matrix metallopeptidase 9 (Gelatinase B) |
| MMP12 | Macrophage metalloelastase |
| MMP7 | Matrilysin |
| MPO | Myeloperoxidase |
| MSLN | Mesothelin |
| MUC_16 | Mucin 16 |
| NCR1 | Natural cytotoxicity triggering receptor 1 |
| NEMO | NF-kappa-B essential modulator |
| NF2 | Neurofibromin 2 |
| NFATC3 | Nuclear factor of activated T-cells, cytoplasmic 3 |
| Notch_3 | Notch homolog 3 |
| NT_proBNP | N-terminal pro B-type natriuretic peptide |
| NTF4 | Neurotrophin 4 |
| nucleotidase_5_NT | 5'-nucleotidase |
| OPG | Osteoprotegerin |
| OPN | Osteopontin |
| PADI2 | Peptidyl arginine deiminase 2 |
| PAI | Plasminogen activator inhibitor |
| PAPPA | Pappalysin-1 |
| PAR_1 | Protease-activated receptor 1 |
| PARP_1 | Poly(ADP-ribose) polymerase 1 |
| PCSK9 | Proprotein convertase subtilisin/kexin type 9 |
| PD_L2 | Programmed cell death ligand 2 |
| PDGF_subunit_A | Platelet-derived growth factor subunit A |
| PDGF_subunit_B | Platelet-derived growth factor subunit B |
| PECAM_1 | Platelet endothelial cell adhesion molecule 1 |
| PGF | Placenta growth factor |
| PGLYRP1 | Peptidoglycan recognition protein 1 |
| PI3 | Elafin |
| PIgR | Polymeric immunoglobulin receptor |
| PIK3AP1 | Phosphoinositide-3-kinase adapter protein 1 |
| PLC | Phospholipase C |
| PLXNA4 | Plexin A4 |
| PODXL | Podocalyxin-like protein |
| PON3 | Serum paraoxonase/lactonase 3 |
| PPP1R9B | Protein phosphatase 1 regulatory subunit 9B |
| PPY | Peptide YY |
| PRDX1 | Peroxiredoxin 1 |
| PRDX3 | Peroxiredoxin 3 |
| PRDX5 | Peroxiredoxin 5 |
| PRELP | Prolargin |
| PRKCQ | Protein kinase C theta |
| PRSS27 | Serine protease 27 |
| PRSS8 | Prostasin |
| PRTN3 | Myeloblastin |
| PSGL_1 | P-selectin glycoprotein ligand 1 |
| PSIP1 | PC4 and SFRS1 interacting protein 1 |
| PSP_D | Serine protease inhibitor D |
| PTH1R | Parathyroid hormone 1 receptor |
| PTX3 | Pentraxin-related protein PTX3 |
| PVRL4 | Poliovirus receptor-related 4 |
| RAGE | Receptor for advanced glycation end products |
| RARRES2 | Retinoic acid receptor responder protein 2 |
| REN | Renin |
| RET | Ret proto-oncogene |
| RETN | Resistin |
| RSPO3 | R-spondin-3 |
| S100A11 | S100 calcium-binding protein A11 |
| S100A4 | S100 calcium-binding protein A4 |
| SCAMP3 | Secretory carrier membrane protein 3 |
| SCF_CVDII | Stem cell factor (CVDII) |
| SCF_oncopanel | Stem cell factor (Oncopanel) |
| SCGB3A2 | Secretoglobin family 3A member 2 |
| SELE | E-selectin |
| SELP | P-selectin |
| SERPINA12 | Serpin A12 |
| SEZ6L | Seizure related 6 homolog like |
| SH2B3 | SH2B adaptor protein 3 |
| SH2D1A | SH2 domain-containing protein 1A |
| SHPS_1 | SH2 domain-containing protein 1 |
| SIT 1.00 | Serotonin transporter |
| SLAMF7 | SLAM family member 7 |
| SOD2 | Superoxide dismutase [Mn], mitochondrial |
| SORT1 | Sortilin |
| SPARC | Secreted protein acidic and cysteine-rich |
| SPON1 | Spondin-1 |
| SPON2 | Spondin-2 |
| SPRY2 | Sprouty homolog 2 |
| SRC | Proto-oncogene tyrosine-protein kinase Src |
| SRPK2 | Serine/arginine-rich protein kinase 2 |
| ST2 | Interleukin 1 receptor-like 1 |
| STC1 | Stanniocalcin 1 |
| STK4 | Serine/threonine-protein kinase 4 |
| SYND1 | Syndecan 1 |
| t_PA | Tissue-type plasminogen activator |
| TANK | TRAF family member-associated NF-kappa-B activator |
| TCL1A | T-cell leukemia/lymphoma 1A |
| TF | Transferrin |
| TFF3 | Trefoil factor 3 |
| TFPI | Tissue factor pathway inhibitor |
| TFPI_2 | Tissue factor pathway inhibitor 2 |
| TGF_alpha | Transforming growth factor alpha |
| TGFR_2 | Transforming growth factor receptor 2 |
| TGM2 | Protein-glutamine gamma-glutamyltransferase 2 |
| THBS2 | Thrombospondin-2 |
| THPO | Thrombopoietin |
| TIE2 | Tyrosine kinase with immunoglobulin-like and EGF-like domains 2 |
| TIMP4 | Metalloproteinase inhibitor 4 |
| TLR3 | Toll-like receptor 3 |
| TLT_2 | T-cell immunoglobulin and mucin-domain containing protein 2 |
| TM | Thrombomodulin |
| TNF_R1 | Tumor necrosis factor receptor 1 |
| TNF_R2 | Tumor necrosis factor receptor 2 |
| TNFRSF10A | Tumor necrosis factor receptor superfamily member 10A |
| TNFRSF10C | Tumor necrosis factor receptor superfamily member 10C |
| TNFRSF11A | Tumor necrosis factor receptor superfamily member 11A |
| TNFRSF13B | Tumor necrosis factor receptor superfamily member 13B |
| TNFRSF14 | Tumor necrosis factor receptor superfamily member 14 |
| TNFRSF19 | Tumor necrosis factor receptor superfamily member 19 |
| TNFRSF4 | Tumor necrosis factor receptor superfamily member 4 |
| TNFRSF6B | Tumor necrosis factor receptor superfamily member 6B |
| TNFSF13 | Tumor necrosis factor superfamily member 13 |
| TNFSF13B | Tumor necrosis factor ligand superfamily member 13B |
| TPSAB1 | Tryptase alpha/beta 1 |
| TR | Thyroid receptor |
| TR_AP | Thyroid receptor, alpha isoform |
| TRAF2 | TNF receptor-associated factor 2 |
| TRAIL | TNF-related apoptosis-inducing ligand |
| TRAIL_R2 | TRAIL receptor 2 |
| TREM1 | Triggering receptor expressed on myeloid cells 1 |
| TRIM21 | Tripartite motif-containing protein 21 |
| TRIM5 | Tripartite motif-containing protein 5 |
| TXLNA | Taxilin alpha |
| U_PAR | Urokinase plasminogen activator receptor |
| uPA | Urokinase plasminogen activator |
| VEGFA | Vascular endothelial growth factor A |
| VEGFD | Vascular endothelial growth factor D |
| VEGFR_2 | Vascular endothelial growth factor receptor 2 |
| VEGFR_3 | Vascular endothelial growth factor receptor 3 |
| VIM | Vimentin |
| VSIG2 | V-set and immunoglobulin domain-containing protein 2 |
| vWF | von Willebrand factor |
| WFDC2 | WAP four-disulfide core domain protein 2 |
| WIF_1 | Wnt inhibitory factor 1 |
| WISP_1 | Wnt-inducible signaling pathway protein 1 |
| XCL1 | Lymphotactin |
| XPNPEP2 | X-prolyl aminopeptidase 2 |
| ZBTB16 | Zinc finger and BTB domain-containing protein 16 |

**Supplementary figure 2:** (A) Plasma CA125 levels between HFpEF patients with no RVD vs RVD

(B) Partial correlation between log-normal transformed CA125 levels and RVEF on CMR

**(A)**  **(B)**

**Supplementary Table 3:** Univariable linear regression analysis between selected variables and log-normalised CA125.

| **Variable** | **Univariable** | | |
| --- | --- | --- | --- |
|  | Std. β | p |  |
| Age | -0.002 | 0.99 |  |
| Male gender | 0.02 | 0.90 |  |
| BMI | 0.012 | 0.38 |  |
| Average E/e' | 0.06 | 0.69 |  |
| RVEF (%) | **-0.34** | **0.004** |  |
| LVEF (%) | -0.21 | 0.082 |  |
| LneGFR | -0.19 | 0.14 |  |
| AF (absent or present) | 0.01 | 0.42 |  |

RVEF: right ventricular ejection fraction; LVEF: left ventricular ejection fraction; LneGFR: log-normalised estimated glomerular filtration rate; AF: atrial fibrillation

**Supplementary Table 4:** Univariable and multivariable linear regression analysis of selected baseline characteristics, clinical and imaging parameters with CMR RV ejection fraction (RVEF)

| **Variables** | **Univariable** | | **Multivariable** | |
| --- | --- | --- | --- | --- |
|  | Std. β | p | Std. β | p |
| Age (years) | **-0.31** | **0.006** | **-0.28** | **0.004** |
| BMI (kg/m^2^) | -0.026 | 0.83 |  |  |
| AF | **-0.50** | **<0.001** | **-0.33** | **0.001** |
| LneGFR | 0.13 | 0.30 |  |  |
| LVEF (%) | **0.45** | **<0.001** | **0.35** | **<0.001** |
| Diuretic use | **-0.29** | **0.01** | NS |  |
| LnCA125 | **-0.34** | **0.004** | **-0.24** | **0.01** |
| LnNTproBNP | **-0.35** | **0.002** | NS |  |

BMI: body mass index; AF: atrial fibrillation; LVEF: left ventricular ejection fraction; LneGFR: log-normalised estimated glomerular filtration rate; LAEF: left atrial emptying fraction; LnCA125: log-normalised carbohydrate antigen 125; LnNTproBNP: log-normalised N-terminal probrain natriuretic peptide. Both backward and forward linear regression analyses performed, with forward linear regression analysis values presented.

**Supplementary Figure 3:** Receiver operator curve analysis of CA125 and NTproBNP in predicting right ventricular dysfunction (CMR RVEF<45%)


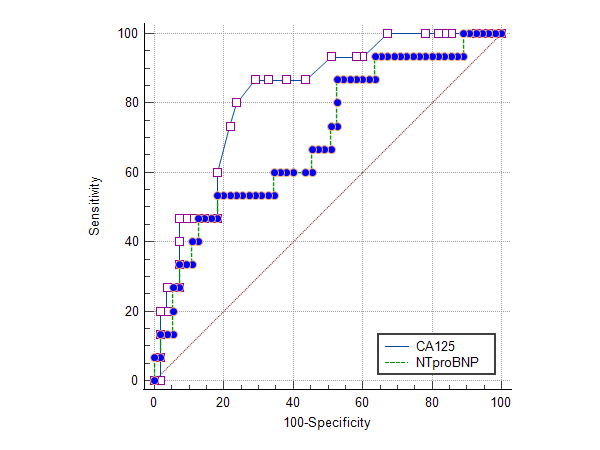


**Supplementary Table 5:** Prediction models for combined outcome of all-cause death and heart failure hospitalisation

|  | **Concordance** | **AIC** | **χ^2^** | **p-value** |
| --- | --- | --- | --- | --- |
| **Model 1 (reference)** |  |  |  |  |
| Age, Gender, BMI, AF, RVD, LVEF, LnNTpro, LnCA125, LneGFR | 0.85 | 150.4 |  |  |
| **Model 2 (LnCA125 removed)** | 0.79 | 157.6 | 9.27 | **0.002** |
| **Model 3 (BMI removed)** | 0.83 | 157 | 8.64 | **0.003** |
| **Model 4 (lneGFR removed)** | 0.84 | 152.3 | 3.89 | **0.049** |

BMI: body mass index (continuous); RVD: right ventricular dysfunction (RVEF<45% on CMR); LVEF: left ventricular ejection fraction (continuous); LnNTproBNP: log-normalised N-terminal probrain natriuretic peptide; LnCA125: log-normalised carbohydrate antigen 125; LneGFR: log-normalised estimated glomerular filtration rate. AIC: Akaike’s information criterion (AIC). χ^2^ : Chi-squared value from likelihood ratio test between subsequent models with model 1 (reference model)

**Supplementary Table 6:** Baseline characteristics, echocardiography and CMR according to median CA125 levels*

|  | **CA125≤ 17kU/L**  **(n=36)** | **CA125> 17kU/L**  **(n=34)** | **p** |
| --- | --- | --- | --- |
| Age, years (SD) | 73 (8) | 74 (8) | 0.62 |
| Female (%) | 19 (53) | 19 (56) | 0.79 |
| BMI, kg/m^2^ (SD) | 30 (6) | 31 (5) | 0.73 |
| **Comorbidities** |  |  |  |
| Hypertension (%) | 30 (83) | 28 (82) | 0.91 |
| Coronary artery disease (%) | 13 (36) | 11 (32) | 0.74 |
| Atrial fibrillation (%) | 23 (67) | 24 (71) | 0.55 |
| Diabetes mellitus (%) | 15 (42) | 15 (44) | 0.84 |
| COPD (%) | 4 (11) | 8 (24) | 0.17 |
| **HF characteristics** |  |  |  |
| NYHA functional class |  |  | **0.03** |
| Class II (%) | 24 (67) | 14 (41) |  |
| Class III (%) | 12 (33) | 20 (59) |  |
| Previous HF hospitalization (%) | 13 (36) | 19 (56) | 0.1 |
| **Medications** |  |  |  |
| Beta-blocker (%) | 31 (86) | 33 (97) | 0.1 |
| ACEi/ARB (%) | 23 (64) | 21 (62) | 0.85 |
| MRA (%) | 15 (42) | 13 (38) | 0.77 |
| Diuretics (%) | 30 (83) | 33 (97) | 0.06 |
| **Laboratory** |  |  |  |
| Hemoglobin, mmol/L (SD) | 8.1 (1.1) | 7.9 (1.2) | 0.58 |
| Hematocrit, % (SD) | 0.40 (0.05) | 0.39 (0.05) | 0.58 |
| C-reactive protein, mg/L (SD) | 4.4 (4.4) | 9.1 (8.9) | **0.03** |
| eGFR, ml/min/1.73m^2^ (SD) | 55 (21) | 48 (20) | 0.17 |
| NTproBNP, ng/L (SD) | 1521 (1396) | 3035 (2754) | **0.006** |
| AST/ALT (SD) | 1.5 (1.3) | 1.3 (0.4) | 0.42 |
| **Clinical Outcomes** |  |  |  |
| All-cause death or HF hospitalization (%) | 7 (19) | 16 (47) | **0.01** |
| All-cause death (%) | 5 (14) | 8 (24) | 0.3 |
| HF hospitalization (%) | 5 (14) | 12 (35) | **0.04** |
| **Echocardiography** |  |  |  |
| LV ejection fraction, % (SD) | 56 (4) | 56 (6) | 0.96 |
| Average E/e' (SD) | 13 (5) | 15 (5) | 0.18 |
| LA volume index, ml/m^2^ (SD) | 45 (17) | 48 (11) | 0.42 |
| TAPSE, mm (SD) | 20 (4) | 21 (4) | 0.69 |
| RV S', cm/s (SD) | 12 (3) | 12 (3) | 0.64 |
| RV FAC, % (SD) | 48 (11) | 47 (14) | 0.81 |
| RV end-diastolic area, cm^2^ (SD) | 15 (6) | 19 (8) | **0.04** |
| SPAP, mmHg (SD) | 37 (16) | 41 (12) | 0.43 |
| TAPSE/SPAP, mm/mmHg (SD) | 0.70 (0.40) | 0.51 (0.20) | 0.13 |
| **CMR** |  |  |  |
| LVEDVi, ml/m^2^ (SD) | 83 (20) | 86 (26) | 0.55 |
| LVEF, % (SD) | 56 (7) | 53 (8) | 0.17 |
| LV mass index, g/m^2^ (SD) | 50 (13) | 62 (28) | **0.02** |
| LV GLS, % (SD) | 17 (6) | 17 (5) | 0.54 |
| RVEDVi, ml/m^2^ (SD) | 77 (17) | 83 (21) | 0.2 |
| RVEF, % (SD) | 56 (10) | 49 (11) | **0.005** |
| RV GLS, % (SD) | 21 (6) | 19 (6) | 0.09 |
| RV SV/ESV (SD) | 1.4 (0.6) | 1.1 (0.6) | **0.03** |
| LAESVi, ml/m^2^ (SD) | 61 (20) | 61 (19) | 0.99 |
| LA emptying fraction, % (SD) | 32 (17) | 23 (14) | **0.02** |
| LA reservoir strain, % (SD) | 16 (9) | 11 (7) | **0.02** |
| LA passive strain, % (SD) | 10 (5) | 9 (5) | 0.62 |
| LA active strain, % (SD) | 9 (6) | 7 (5) | 0.35 |
| RAESVi, ml/m^2^ (SD) | 41 (18) | 50 (24) | 0.08 |
| RA emptying fraction, % (SD) | 32 (17) | 25 (16) | 0.07 |
| RA reservoir strain, % (SD) | 23 (16) | 17 (12) | 0.07 |
| RA passive strain, % (SD) | 12 (6) | 11 (8) | 0.93 |
| RA active strain, % (SD) | 17 (11) | 18 (7) | 0.78 |

**CA125 levels available for 70 of 77 patients*

BMI: body mass index; BP: blood pressure; COPD: chronic obstructive pulmonary disease; NYHA: New York Heart Association; ACEi: angiotensin converting enzyme inhibitor; ARB: angiotensin receptor blocker; MRA: mineralocorticoid receptor antagonist; eGFR: estimated glomerular filtration rate; NT-proBNP: N-terminal probrain natriuretic peptide; AST/ALT: aspartate aminotransferase/alanine aminotransferase ratio; LV: left ventricle; LA: left atrium TAPSE: tricuspid annular plane systolic excursion; RV: right ventricle; FAC: fractional area change; SPAP: pulmonary artery systolic pressure; EDVi: end-diastolic volume index; ESVi: end-systolic volume index; GLS: global longitudinal strain; RV SV/ESV: right ventricular stroke volume/end-systolic volume ratio; LAESVi: left atrial end-systolic volume index; RAESVi: right atrial end-systolic volume index. Mean values and standard deviations presented.
